# *FGFR1* variants contributed to families with tooth agenesis

**DOI:** 10.1186/s40246-023-00539-8

**Published:** 2023-10-13

**Authors:** Siyue Yao, Xi Zhou, Min Gu, Chengcheng Zhang, Oliver Bartsch, Barbara Vona, Liwen Fan, Lan Ma, Yongchu Pan

**Affiliations:** 1 Department of Orthodontics, The Affiliated Stomatology Hospital of Nanjing Medical University, Nanjing, China; 2 Jiangsu Province Key Laboratory of Oral Diseases, Nanjing Medical University, 136 Hanzhong Road, Nanjing, 210029 China; 3 The Affiliated Stomatology Hospital of Suzhou Vocational Health College, Suzhou, 215000 China; 4 Department of Stomatology, Affiliated Third Hospital of Soochow University, The First People’s Hospital of Changzhou City, Changzhou City, 213003 Jiangsu Province China; 5 Institute of Human Genetics, University Medical Centre of the Johannes Gutenberg University Mainz, Mainz, Germany; 6 Institute of Human Genetics, University Medical Center Göttingen, Göttingen, Germany; 7 Institute for Auditory Neuroscience and InnerEarLab, University Medical Center Göttingen, Göttingen, Germany; 8 Jiangsu Province Engineering Research Center of Stomatological Translational Medicine, Nanjing Medical University, Nanjing, China

**Keywords:** Tooth agenesis, Whole-exome sequencing, *FGFR1*, Genetic research, Developmental biology

## Abstract

**Background:**

Tooth agenesis is a common dental anomaly that can substantially affect both the ability to chew and the esthetic appearance of patients. This study aims to identify possible genetic factors that underlie various forms of tooth agenesis and to investigate the possible molecular mechanisms through which human dental pulp stem cells may play a role in this condition.

**Results:**

Using whole-exome sequencing of a Han Chinese family with non-syndromic tooth agenesis, a rare mutation in *FGFR1* (NM_001174063.2: c.103G > A, p.Gly35Arg) was identified as causative and confirmed by Sanger sequencing. Via GeneMatcher, another family with a known variant (NM_001174063.2: c.1859G > A, p.Arg620Gln) was identified and diagnosed with tooth agenesis and a rare genetic disorder with considerable intrafamilial variability. *Fgfr1* is enriched in the ectoderm during early embryonic development of mice and showed sustained low expression during normal embryonic development of *Xenopus laevis* frogs. Functional studies of the highly conserved missense variant c.103G > A showed deleterious effects. *FGFR1* (c.103G > A) was overexpressed compared to wildtype and promoted proliferation while inhibiting apoptosis in HEK293 and human dental pulp stem cells. Moreover, the c.103G > A variant was found to suppress the epithelial-mesenchymal transition. The variant could downregulate ID4 expression and deactivate the TGF-beta signaling pathway by promoting the expression of SMAD6 and SMAD7.

**Conclusion:**

Our research broadens the mutation spectrum associated with tooth agenesis and enhances understanding of the underlying disease mechanisms of this condition.

**Supplementary Information:**

The online version contains supplementary material available at 10.1186/s40246-023-00539-8.

## Background

Tooth agenesis (TA), a congenital disorder of tooth development, is characterized by missing teeth and mainly caused by genetic factors, and in a subset of cases, by environmental factors [1]. Both deciduous and permanent dentition can be affected. TA in deciduous dentition has a prevalence of less than 1% in European populations but is higher in Asian populations [2, 3]. Moreover, agenesis of primary teeth is often accompanied by agenesis of inherited permanent teeth [4]. The prevalence of TA in permanent dentition is significantly higher at 5.89% in Chinese [5] and 5.5% in Europeans [6], than that in deciduous dentition.

Tooth development is a complex biological process spanning from embryonic age to postnatal development and includes cell–cell and epithelial–mesenchymal interaction, cell differentiation, morphogenesis, tissue mineralization and tooth eruption [7]. Environmental factors like trauma, infection and toxins may affect proliferation and migration of neural crest cells, which can differentiate into teeth [8]. Genetic factors play an important role in tooth development and numerous genes are associated with non-syndromic tooth agenesis (NSTA), including *AXIN2*, *EDA*, *LRP6*, *MSX1*, *PAX9*, *WNT10A* and *WNT10B* [9]. In 2015, whole-exome sequencing (WES) studies in 20 unrelated individuals indicated that loss-of-function mutations in the *WNT* co-receptor *LRP6* could cause autosomal dominant oligodontia [10]. Two studies have proposed *TSPEAR* as a novel NSTA-related gene [11, 12]. These and other findings indicate that a series of genetically controlled successive molecular interactions, including the fibroblast growth factor (Fgf), wingless-related integration site (Wnt), bone morphogenic protein (Bmp) and hedgehog (Hh) pathways, take part in the signaling of epithelial-mesenchymal interactions and are involved in the development of teeth [13–15]. Recently, a single-cell interactome study of human tooth germ from the growing third molar added proof that BMP, FGF and MSX1 comprise a network of tooth development regulators [16].

Here, we recruited a Han Chinese family and matched with a German family to investigate genetic variants underlying TA. Functional studies showed that the c.103G > A variant in *FGFR1* from the Han Chinese family was associated with increased gene expression, reduced cell apoptosis and promoted proliferation, and thus affected the epithelial-mesenchymal transition (EMT) conversion process by inhibiting the downstream TGF-beta signaling pathway.

## Materials and methods

### Recruitment of pedigrees

**Fig. 1:**
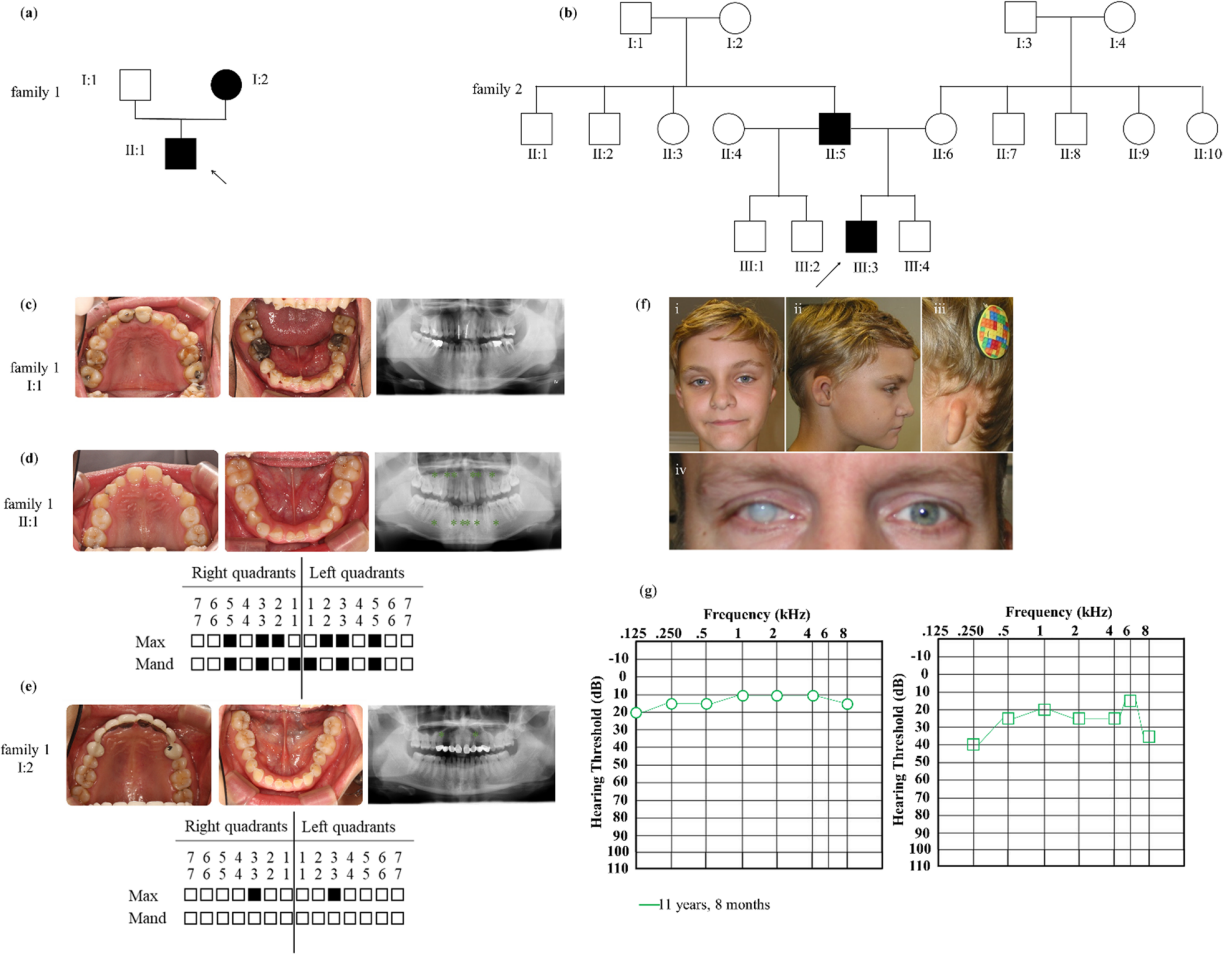
Pedigrees and phenotypes in the families. **a** Pedigree of family 1. The proband (II:1) is a 9-year-old boy with NSTA. **b** Pedigree of family 2. The proband (III:3) is an 11-year-old boy with agenesis of eight permanent teeth, auricular dysplasia, hearing impairment and further findings of olfactory dysfunction. Square, male; circle, female; black, patient; arrow, proband. **c**–**e** Intra-oral photographs and panoramic radiographs of individuals from family 1. Schematic of congenitally missing teeth of proband and his mother. Asterisks and solid squares indicate the congenitally missing teeth. Max, maxillary; Mand, mandibular. **f** Photographs of the proband and father in family 2. (i) Frontal view, (ii) right profile and ear and (iii) left profile and ear. First- and third-degree microtia of the right and left ear, respectively. (iv) Mature cataract in the right eye of the father. **g** Pure-tone audiometry of the proband in family 2 following bone-conduction implantation, note normal hearing on the right ear and mild hearing loss on the left ear

This study included two families, family 1 from China (Fig. 1a) and family 2 from Germany (Fig. 1b) who matched from GeneMatcher based on a common candidate gene [17]. Family 1 from China consisted of three individuals across two generations, with the nine-year-old proband diagnosed by orthodontists from the Affiliated Stomatology Hospital of Nanjing Medical University using panoramic radiography and clinical examination. All participants in family 1 have no syndromes and supernumerary teeth. Family 2 from Germany underwent expert clinical examination at the University Medical Centre of Mainz.

### Molecular genetic testing

**Fig. 2:**
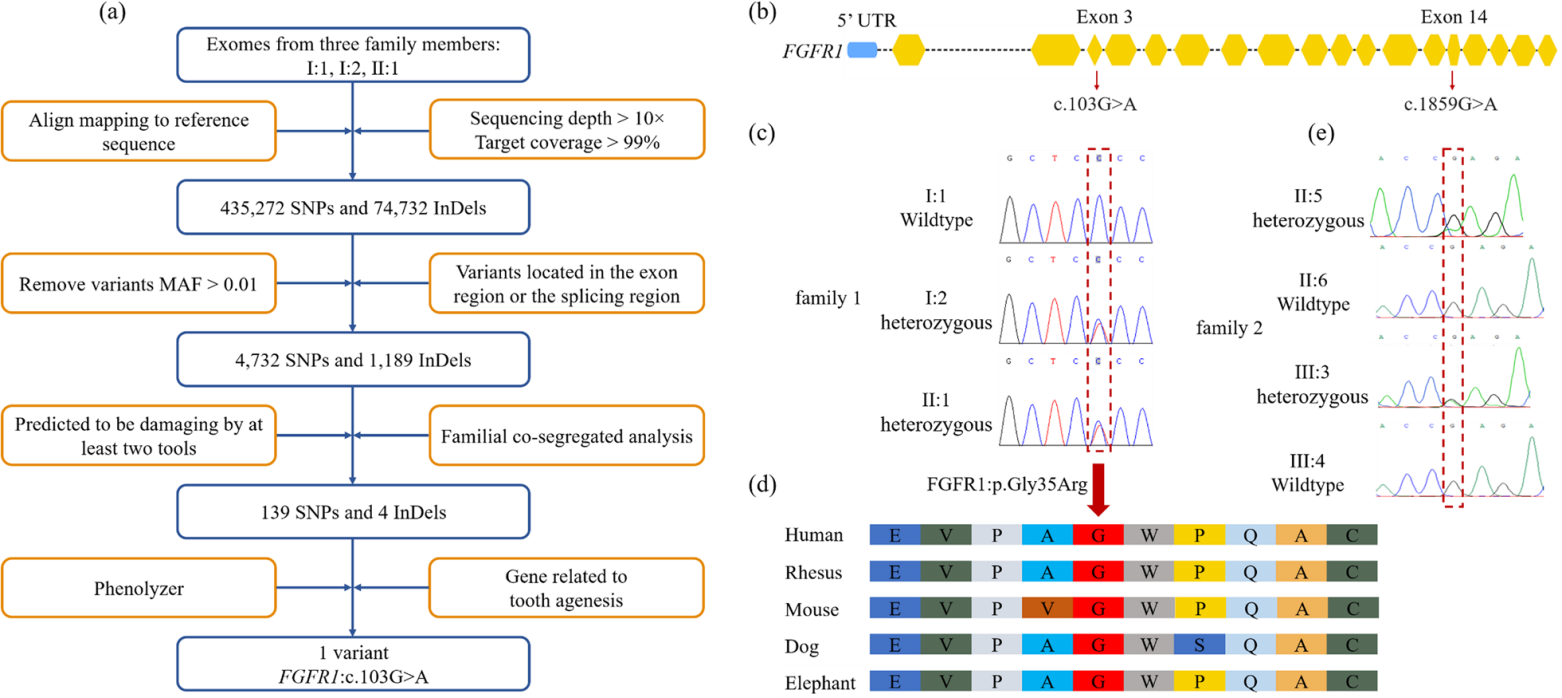
**a** Flow chart outlining selection of the causative variant. **b** Schematic diagram of the gene location of the damaging allele. **c** Sanger sequencing of the heterozygous c.103G > A (I:2 and II:1) and wildtype (I:1) alleles in the *FGFR1* gene. Red dotted frames indicate the positions of causative variants. **d** Conservation of each amino acid residue across species is shown. The red arrow indicates the mutated amino acid. Glycine at position 35 is conserved. **e** Sanger sequencing of the heterozygous c.1859G > A (II:5 and III:3) and wildtype (II:6 and III:4) alleles in the *FGFR1* gene. Red dotted frame indicates the position of causative variants

In family 1, genomic DNAs from all family members were extracted using a Qiagen Blood Kit (Qiagen, Hilden, Germany) and whole-exome capture was performed with the Agilent SureSelect Human All Exon V6 followed by next-generation sequencing on an Illumina HiSeq sequencer. Variant analysis was performed with the Genome Analysis Toolkit (GATK, version 3.3.0). Multi-sample variant calling was performed by HaplotypeCaller, and variants were filtered by Variant Quality Score Recalibration for both single nucleotide polymorphisms (SNPs) and insertions/deletions (InDels) with the following filters: (1) removal of Exome Aggregation Consortium (ExAC), 1,000 Genomes Project and Exome Sequencing Project (ESP6500) browser variants with a minor allele frequency (MAF) > 0.01, (2) retaining variants located in the exon and splicing regions, (3) retaining SNPs predicted to be harmful by at least two tools (SIFT, PolyPhen-2, MutationTaster and CADD), (4) keeping variants common in all patients but not in normal subjects, (5) identifying the potential causative variant related to TA which was predicted by Phenolyzer (Fig. 2a).

For family 2, genomic DNAs from the proband, his parents and younger brother were extracted from whole blood using an automated standard procedure. A custom-designed targeted genomic panel including 151 syndromic and non-syndromic deafness genes was performed as previously described [18]. All variants were mapped to the human reference sequence GRCh37/hg19.

### Sanger sequencing

The variants in both families were validated using Sanger sequencing. Primers of *FGFR1* were designed (family 1: forward: 5’-AAACATTGACGGAGAAGTAGGTG-3’; reverse: 5’-TTCCTAACTTTGCCTCTTTCTTC-3’, family 2: forward: 5’-CTAGTTGCATGGGTGGCG-3’; reverse: 5’-GTTCTCAGCCCACCCCAC-3’) with Primer-BLAST (NCBI). In family 1, the Sanger sequencing data were analyzed using Chromas (version 1.0.0.1, Technelysium Pty Ltd., Australia). In family 2, the Sanger sequencing data were analyzed using Mutation Surveyor. The variants co-segregated with disease in the two families.

### Cell culture, lentiviral construction and transfection

Human embryonic kidney 293 (HEK293; ATCC^CRL-1573^) were purchased from ATCC and human dental pulp stem cells (hDPSCs) were isolated from tooth extraction. Pulp tissues were minced, digested with collagenase type I (Item#: 1904MG100, BioFroxx, Germany) and trypsin with alpha-modified minimum essential medium Eagle (α-MEM) in a centrifuge tube with shaking every 5 min for 4 times and collected in a medium-sized dish. The third generation of dental pulp stem cells (DPSCs) were harvested and incubated with the antibodies CD29-APC, CD90-FITC, CD73-PE and CD45-PE (BD Pharmingen, England) for 1 h in the dark and washed twice with PBS. The specific fluorescence of the samples was examined with a flow cytometer (BD Biosciences, San Jose, CA, USA). HEK293 and hDPSCs were cultured in Eagle's minimum essential medium (EMEM) and α-MEM supplemented with 10% fetal bovine serum, 1% penicillin–streptomycin solution at 37 °C in 5% CO_2_.

Lentiviruses (Lenti-*FGFR1*-MT-G103A-3FLAG-OE and Lenti-*FGFR1*-3FLAG-OE) were prepared by transfection of plasmids containing the open reading frame of wildtype or mutant human *FGFR1* into HEK293. Lentivirus (FV115-mCMV-ZsGreen) was used as a control. When HEK293 and hDPSCs reached 40–50% confluency, they were transfected with three lentiviruses (Lenti-*FGFR1*-MT-G103A-3FLAG-OE; Lenti-*FGFR1*-3FLAG-OE and FV115-mCMV-ZsGreen) at a multiplicity of transfection of 5 pfu/HEK293 and 100 pfu/hDPSC in the medium containing 5 μg/mL polybrene.

### RNA extraction and quantitative real-time polymerase chain reaction (qRT-PCR)

To compare gene expression of cells which were transfected by lentiviruses, total RNA was extracted from the cells by FastPure® Cell/Tissue Total RNA Isolation Kit V2 (Vazyme, Nanjing, China) and reverse-transcribed to cDNA (RR036A, Takara Bio, Shiga, Japan). The mRNA expression was evaluated using SYBR Mastermix (Q712-02, Vazyme, Nanjing, China) on QuantStudio7 qRT-PCR System (Applied Biosystems) and normalized against the endogenous *GAPDH* RNA control. The primers were listed as follows: *FGFR1* (forward: 5’-ACGCAGGATGGTCCCTT-3’, reverse: 5’-GTTGTGGCTGGGGTTGTAG-3’) and *GAPDH* (forward: 5’GGACCTGACCTGCCGTCTAG-3’, reverse: 5’-GTAGCCCAGGATGCCCTTGA-3’).

### Immunofluorescence (IF) staining

The cells which were transfected with lentiviruses were fixed with 4% paraformaldehyde, infiltrated with Triton X-100 solution (Beyotime, China) for 12 min and blocked with goat serum. Then, cells were washed by PBS twice and incubated with anti-FGFR1 antibody (diluted 1:1000, CST, #9740) overnight, followed by incubation with a mixture of secondary antibody with fluorochrome for 1.5 h in the dark. The nuclei were stained with DAPI (Beyotime, China). Finally, the result was observed under a fluorescence microscope (Leica, Germany).

### Western blot

To evaluate the expression of target proteins of transfected cells, we collected the cells for lysis in RIPA buffer (Beyotime, Shanghai, China) on ice. Protein samples of the same amount were loaded in 10% SDS-PAGE for electrophoresis separation and transferred to 0.22 μm polyvinylidene difluoride (PVDF) membranes (Millipore, Massachusetts, USA). While blocked with 5% non-fat milk for 2 h at room temperature, the membranes were incubated at 4 °C overnight with primary antibodies including FGFR1 (diluted 1:1000, CST, #9740), E-cadherin (diluted 1:1000, CST, #3195), N-cadherin (diluted 1:1000, CST, #13,116), Vimentin (diluted 1:1000, CST, #5741), ID4 (diluted 1:1000, Abcam, ab220166), SMAD6 (diluted 1:1000, ab273106), SMAD7 (diluted 1:1000, ab216428) and GAPDH (diluted 1:1000, Beyotime, AG019). When washed with Tris-buffered saline containing 0.05% Tween-20 (TBST buffer) for three times and incubated with horseradish peroxidase-conjugated secondary antibodies (1:10,000), the protein bands were visualized by chemiluminescence reagents (P10100, NcmECL Ultra).

### Cell apoptosis and proliferation assay

For determination of apoptosis, transfected cells were seeded in six-well plates, treated with trypsin (Gibco, Grand Island, USA) and resuspended as a single-cell suspension after incubating 48 h. We used Annexin V-PE Apoptosis Detection Kit (BD Biosciences, San Jose, CA, USA) to stain cells and analyzed using a Fluorescence Activated Cell Sorting (FACS) System by BD Biosciences (San Jose, CA, USA). Data were analyzed with FlowJo software (TreeStar, Ashland, OR, USA). Cell proliferation was assessed by absorbance using a Cell Counting Kit-8 assay (CCK8, Dojindo, Kumamoto, Japan) according to the manufacturer’s instructions. Cells were seeded in 96-well plates at a density of 3 × 10^3^ cells per well. We added 10 μl CCK8 solution into each well for 2 h incubation at 37 °C. The absorbance at a wavelength of 450 nm was measured on a spectrophotometer microplate reader (Multiskan MK3, Thermo).

### RNA sequencing (RNA-seq)

Three pairs of biological replicates (transfected with overexpression lentivirus or control vector as 1 sample) of the RNA sample were collected by 1 mL TRIzol reagent (Invitrogen Corporation). The library preparation was carried out according to the instructions provided with the Trio RNA-Seq Library Preparation Kit (Nugen Technologies, USA) followed by sequencing using an Illumina HiSeq sequencing platform. Skewer software was employed to filter low-quality reads and obtain high-quality clean reads. FastQC software (v0.11.5, http://www.bioinformatics.babraham.ac.uk/projects/fastqc) was employed for quality control analysis. For alignment, STAR software (2.5.3a, https://github.com/alexdobin/STAR) was used to compare the clean reads and the reference gene sequence. Compared against the reference genome, the number of sequences of each chromosome were counted, and then the average depth was calculated within each 5 kb of the reference genome and was taken log_2_ to complete the reference genome density distribution statistics. For all samples, StringTie software (v1.2.1c, http://ccb.jhu.edu/software/stringtie) was used to count the original sequence counts of known genes, and the expression of known genes was calculated using fragments per kilobase million to calculate the metrics.

Differentially expressed transcripts were identified using DEGseq package in BioConductor (https://bioconductor.org/packages/release/bioc/html/DESeq2.html). The absolute value of log_2_fold changes ≥ 1 and *p* ≤ 0.05 of differential genes were integrated to create a volcano plot and heat map. The Kyoto Encyclopedia of Genes and Genomes (KEGG) terms were identified in differentially expressed genes by R Cluster Profiler package (4.0.5).

### Statistical analysis

The GO and KEGG analyses were performed using R software (version 4.0.5), and the false discovery rate was used to control for multiple testing. For all graphs, statistical analyses were performed using a two-tailed, unpaired Student’s *t*-test (GraphPad Prism-6 software; San Diego, CA, USA). Data were considered statistically significant at *p*-values < 0.05.

## Results

### Clinical features

The proband in family 1 (Fig. 1a, II:1) had the most severe phenotype with the absence of 12 permanent teeth, including two maxillary lateral incisors, four canines, two mandibular central incisors and four second premolars (Fig. 1e). His mother had a milder phenotype with congenital absence of only two permanent teeth, the maxillary canines (Fig. 1d). His father’s right maxillary premolar had been extracted due to ectopic eruption decades ago (Fig. 1c). None of the family members had any craniofacial congenital anomalies.

Family 2 included the proband (III:3) and father (II:5) (Fig. 1b) with eight and six congenitally missing permanent teeth, respectively. The proband also had mild hypertelorism, a relatively large mouth, auricular dysplasia (Fig. 1fi-iii), hearing impairment (Fig. 1g) and learning disabilities, while his father suffered from vision loss (Fig. 1f iv).

Compared with the phenotypes of the diseases listed in Additional file 2: Table S2, the members in family 1 were diagnosed with NSTA, while the members in family 2 were diagnosed with Goldenhar syndrome.

### Characterizing the genetic susceptibility of tooth agenesis

After performing WES analysis on the members of family 1, a total of 510,004 unique variants were identified, of which 49,296 were retained as rare variants (MAF < 0.01). Variants present in affected individuals I:2 and II:1 were filtered against those present in unaffected individual I:1. Subsequent ACMG variant classification of variants in exonic and splicing regions was performed, resulting in 139 SNPs and four InDels (Fig. 2a). Among these, a heterozygous missense variant (NM_001174063.2:c.103G > A) in exon 3 of *FGFR1* (Fig. 2b) was most likely predicted to be related with TA by Phenolyzer (http://phenolyzer.glab.org/) [19] and estimated as deleterious in silico by MutationTaster (score = 1, deleterious) and CADD (score = 21.3, deleterious). The variant was present in a heterozygous state in eight individuals in gnomAD (Additional file 1: Table S1).

The proband (II:1) and his mother (I:2) in family 1 were heterozygous, while the unaffected father was wildtype (Fig. 2c). The mutation in *FGFR1* leads to a Glycine-to-Arginine substitution (NP_001167534.1: p.Gly35Arg) in an evolutionarily conserved domain. In addition, this G nucleotide and the Gly amino acid residue are highly conserved across most vertebrates, including human, rhesus monkey, mouse, dog and chicken by multiple-sequence alignment of *FGFR1* (Fig. 2d).

Following gene panel sequencing and analysis, both the proband in family 2 and his father were identified with a heterozygous pathogenic variant (NM_001174063.2: c.1859G > A, p. Arg620Gln) in *FGFR1* (Fig. 2e) exon 14, which is a known Kallmann syndrome type 2 pathogenic variant (KAL2, OMIM #147,950) [20]. Of note, this is the same variant that was previously published as NM_023110.2: c.1865G > A, p. Arg622Gln in KAL2 patients with variable phenotypic expressivity [21, 22]. Possible mosaicism was analyzed in the father and son using PCR and pyrosequencing with normal results (data not shown). Segregation analysis of the variant confirmed wildtype alleles in the unaffected mother and younger brother. The father’s parents were not available for testing, and therefore, we could not determine whether the variant was inherited or arose de novo.

Overall, it is remarkable that the clinical presentation, particularly of the proband in family 2 due to the c.1859G > A variant, is on the severe end of the *FGFR1* mutation spectrum, while the clinical presentation in family 1 with the c.103G > A variant was mild; however, both variants are associated with TA.

### In silico expression of FGFR1 during early embryonic development

Through data query, it was found that *Fgfr1* is enriched in the ectoderm during the early embryonic development of mice (Additional file 1: Fig. S1a), while it has low expression in the maxillary arch of embryonic mice at day 10.5 (http://www.informatics.jax.org/) (Additional file 1: Fig. S1b). Similarly, *fgfr1* is widely expressed in the ectoderm during early embryonic development of *Xenopus laevis* frogs but has low global expression (https://monsoro-lab-ectomap.shinyapps.io/ EctoMAP/) (Additional file 1: Fig. S1c) and tended to be constant during embryonic development (Additional file 1: Fig. S1d).

### Functional effects of the c.103G > A variant on FGFR1 expression, cell proliferation, apoptosis and subcellular localization

**Fig. 3:**
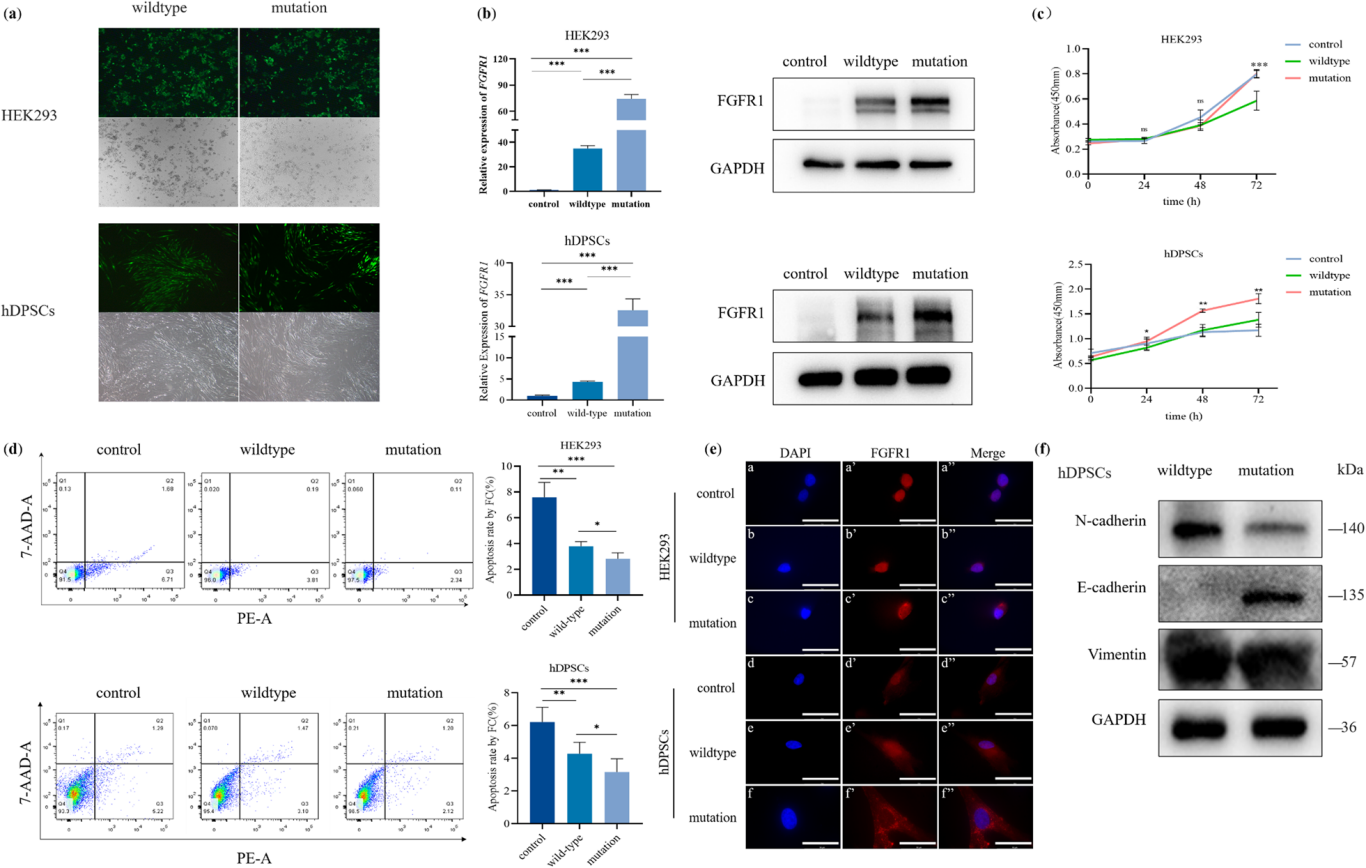
The variant *FGFR1* (c.103G > A) promotes the expression of *FGFR1* and proliferation, reduces apoptosis and affects the process of EMT in HEK293 and hDPSCs. **a** The fluorescence image shows the transfection efficiency of lentiviruses. **b** Protein and mRNA levels of FGFR1 were detected after transfection with *FGFR1* overexpression lentivirus (control, wildtype and mutation). **c** Cell counting kit-8 assay was used to assess the proliferation after transfection with lentivirus (control, wildtype and mutation). **d** Quantitative analysis of cell apoptosis by flow cytometry between the three groups (control, wildtype and mutation). **e** Subcellular localization of wildtype or mutated FGFR1 in the HEK293 and hDPSCs. a'-f' Nuclei stanning by DAPI. b"-h" Merge of FGFR1 and DAPI. *FGFR1* (red); nuclei (blue). Scale bars: 50 μm. **f** Protein levels of EMT markers E-cadherin, N-cadherin and Vimentin in the cells transfected by wildtype and mutation lentivirus were assayed by western blot

As c.103G > A is rare, we performed a series of functional experiments for this variant. The flow cytometric identification of the extracted cells revealed stem cell characteristics of the hDPSCs (Additional file 1: Fig. S2a–d). We generated stably transfected HEK293 and hDPSCs with wildtype and mutant *FGFR1* lentivirus, achieving more than 90% fluorescence efficiency. Expression of *FGFR1* was significantly increased by the mutation c.103G > A in mRNA and protein levels (Fig. 3a and b).

To further investigate the function of c.103G > A (p.Gly35Arg) in *FGFR1*, we performed flow cytometry detection and CCK8 assays that showed that mutation in *FGFR1* significantly promoted cell proliferation (Fig. 3c). Flow cytometric analysis revealed a significantly decreased apoptosis rate in HEK293 and hDPSCs transfected with the mutant allele (Fig. 3d).

To evaluate the subcellular localization of the *FGFR1* c.103G > A variant, we performed immunofluorescence experiments on cells transfected with lentivirus that showed both the wildtype and mutant FGFR1 with a diffuse distribution in the nucleus and cytoplasm. However, FGFR1 protein was retained in the nucleus and the mutant protein was more strongly expressed in the cytoplasm (Fig. 3e).

### Mutation of FGFR1 promotes the process of mesenchymal-epithelial transition

During tooth development, stem cells such as Hertwig's epithelial root sheath can differentiate into cementoblasts through EMT and then induce differentiation of dental papilla to odontoblasts through epithelial-mesenchymal interaction [23]. To understand the impact of the c.103G > A variant on this process, we tested E-cadherin, N-cadherin and Vimentin indicators representing the EMT differentiation process. Our findings showed that the c.103G > A variant led to the upregulation of the epithelial marker E-cadherin and downregulation of mesenchymal markers N-cadherin and Vimentin in the hDPSCs. These results indicated that the *FGFR1* c.103G > A variant may induce the transformation of the mesenchyme to epithelium (Fig. 3f).

### Negative regulation of FGFR1 on TGF-beta signaling pathway

**Fig. 4:**
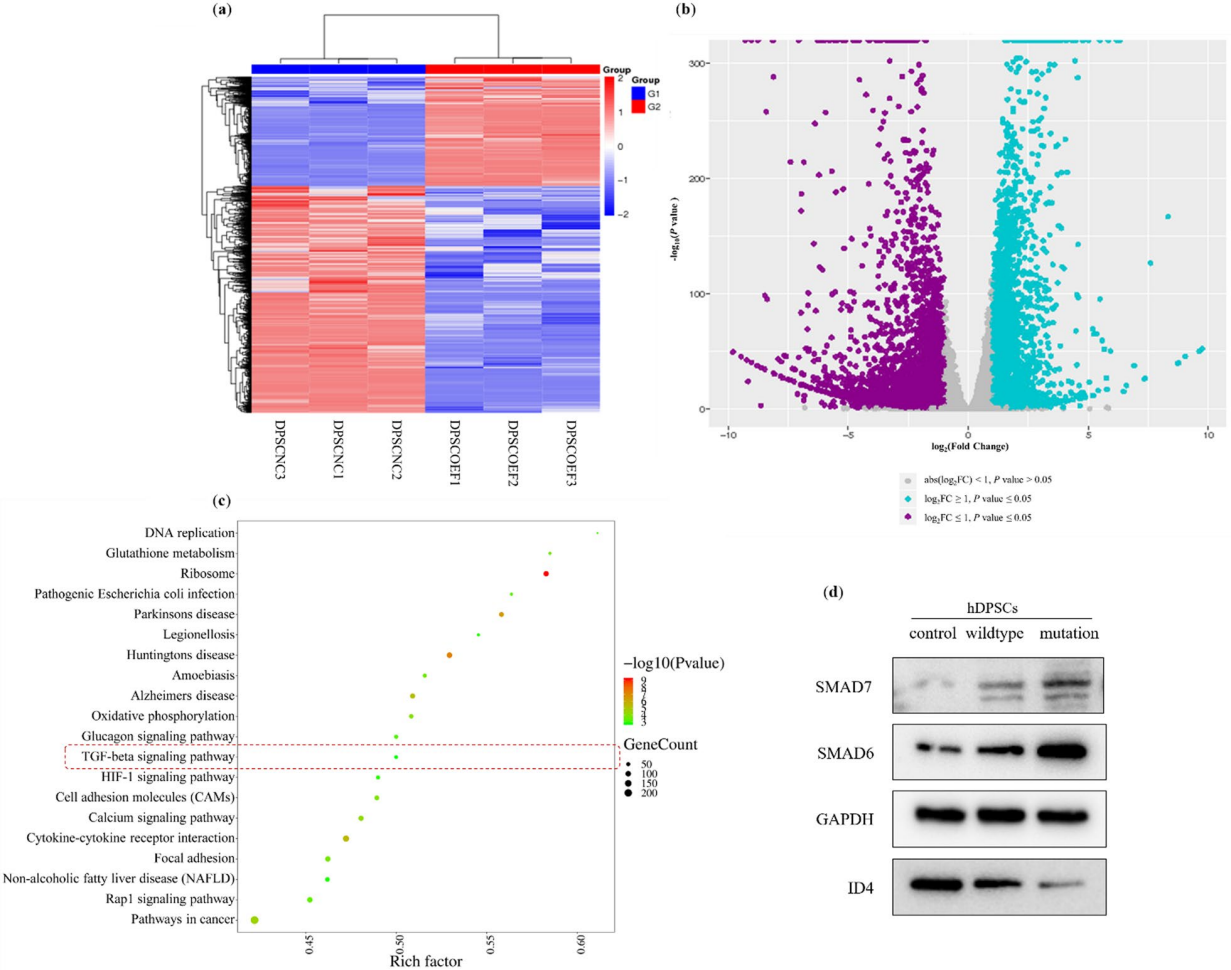
**a** Heatmap showing the expression levels of transcripts in samples from the *FGFR1* overexpression and control groups. Red color refers to upregulation, and blue color refers to downregulation of gene transcription. **b** Volcano plot of differentially expressed genes. Purple dots represent down-regulated genes, and turquoise dots represent up-regulated genes. **c** KEGG pathway analysis ranked the top 20 KEGG pathways (|log_2_FC|≥ 1 and *p* ≤ 0.05). **d** Protein levels of pathway genes were assayed in the cells transfected by control, wildtype and mutation lentivirus by western blot

To investigate the function of *FGFR1* during early human tooth development, we performed RNA-seq on hDPSCs with *FGFR1* overexpression. The RNA-Seq analysis included investigation of reads and comparison of 34,020 differential transcripts (|log_2_FC|≥ 1 and *p* < 0.05), including 21,332 downregulated genes and 12,688 upregulated genes (Fig. 4a and b). We evaluated the screened genes according to their biological function by subjecting these differential genes to KEGG analysis (Fig. 4c) and found that the TGF-beta signaling pathway was among the top 20 pathways affected by *FGFR1* overexpression. The TGF-beta signaling pathway is critical for tooth cell differentiation and EMT [23–25]. Through further analysis of genes in TGF-beta signaling pathway, we found that overexpression of FGFR1 resulted in elevated expression of SMAD6 and SMAD7 and decreased expression of ID4 (Fig. 4d). Previous studies have identified SMAD6, SMAD7 and ID4 as key proteins that inhibit the TGF-beta signaling pathway [25–28]. When SMAD6 and SMAD7 are elevated by overexpression of FGFR1, the TGF-beta signaling pathway is inhibited and results in tooth agenesis [29–31].

## Discussion

By compilation of *FGFR1* mutations described in public databases and the literature, Rivera et al. found that *FGFR1* variants associated with a variety of pathologies were fairly evenly distributed along the entire gene [32]. In cranio-maxillofacial development, *FGFR1* may cause many craniofacial abnormalities, like craniosynostosis [33], abnormal development of the ear and eye [34], tooth agenesis [22, 35–38] and cleft palate [39]. The relevant syndromes and their phenotypes are listed in Additional file 2: Table S2. During tooth development, *FGFR1* is expressed in the ameloblasts and odontoblasts in the mouse [40] and persists in dental epithelial and mesenchymal cells during the development of human primary dentition [41]. In *K14-Cre; Fgfr1*^*fl/fl*^ mice, enamel structure was compromised before tooth eruption [42]. Nevertheless, its function related to tooth development is still unclear.

Here, we describe a heterozygous missense variant (NM_001174063.2: c.103G > A) in *FGFR1* and a known variant (NM_001174063.2: c.1859G > A) as causal for TA. Interestingly, the c.1859G > A (previously reported as NM_023110.2: c.1865G > A) was identified in two unrelated probands with severe microtia and tooth agenesis [21]. Further attempts at a genotype–phenotype correlation revealed a highly variable phenotypic expression which we also observed in family 2. Together with our proband, we therefore recommend consideration of *FGFR1* genetic testing in patients presenting a Goldenhar-like syndrome or microtia to aid in diagnosis and expand the mutation spectrum of *FGFR1*-associated TA.

In the developing tooth, EMT is crucial to induce disengaged odontogenic epithelial stem cells forming supernumerary teeth [43]. E-cadherin and N-cadherin, two significant indicators of EMT, are crucial in the development and shaping of the dental organ [44]. Coordinated expression of E-cadherin and N-cadherin in ameloblasts was significant for secretion of the enamel matrix [44], while abnormal E-cadherin expression and signaling can result in irregular formation of craniofacial structures including teeth [45]. Our study supports that the c.103G > A variant in *FGFR1* enhances gene expression, promotes cell proliferation and reduces apoptosis compared to wildtype. Moreover, the c.103G > A variant may affect the process of EMT conversion due to an increase in the expression of epithelial-related indicators (E-cadherin) and a decrease in mesenchymal-related indicators (N-cadherin) in hDPSCs.

We demonstrate that the *FGFR1* c.103G > A variant not only causes overexpression of FGFR1 but also can affect its proliferation and apoptosis, and can alter its cellular sublocalization. Furthermore, the variant also inhibited TGF-beta signaling by promoting the expression of SMAD6 and SMAD7. Previous studies have identified SMAD6 and SMAD7 as key proteins that inhibit the TGF-beta signaling pathway [25, 26]. Additionally, we demonstrated that overexpression of *FGFR1* downregulates *ID4* expression and thereby inhibits the TGF-beta signaling pathway. As a key gene in the TGF-beta signaling pathway, *ID4* plays a unique role in the EMT process of salivary gland, kidney and lung [3]. Moreover, interaction of TGF-beta and EMT signaling was found to be critical for tooth formation [23, 46]. Therefore, a reduction of TGF-beta signaling and influence of EMT resulting from overexpressed *FGFR1* might underlie TA seen in affected patients.

In summary, we have expanded the mutation and phenotypic spectrum of *FGFR1*-associated TA and proposed possible mechanisms for a nonsynonymous variant (NM_001174063.2: c.103G > A) involved in isolated TA. Unilateral (and occasionally bilateral) microtia and hemifacial microsomia most commonly represents the oculo-auriculo-vertebral spectrum, alias Goldenhar syndrome, which is suspected to be a heterogeneous multifactorial disorder so that molecular genetic diagnostic testing is usually not performed in these patients. However, in patients with bilateral microtia, molecular genetic diagnostic testing is warranted for the identification of a number of monogenic syndromes such as Treacher Collins, Townes-Brocks, CHARGE, Wildervanck and Branchio-oto-renal syndromes, and this report supports that the *FGFR1* c.1859G > A (formerly known as c.1865G > A) variant could be another (rare) cause of Goldenhar-like bilateral microtia. Further studies are warranted to replicate our findings.

## Conclusion

This study highlights how variants in *FGFR1* can lead to vastly different clinical outcomes, demonstrating the diverse clinical spectrum due to *FGFR1* variants.

### Supplementary Information

**Additional file 1**. **Table S1.** The top five candidate variants identified in family 1. **Figure S1.** Gene expression studies of FGFR1. **Figure S2.** Flow cytometric identification of hDPSCs surface markers shows stem cell characteristics.

**Additional file 2**. **Table S2. **The phenotypes with FGFR1 related disorders.

## Data Availability

Data and materials are available on request from the corresponding author.

## Abbreviations

TA: Tooth agenesis
WES: Whole-exome sequencing
Fgf: The fibroblast growth factor
Wnt: Wingless-related integration site
Bmp: Bone morphogenic protein
Hh: Hedgehog
EMT: Epithelial-mesenchymal transition
SNPs: Single nucleotide polymorphisms
InDels: Insertions/deletions
ExAC: Exome Aggregation Consortium
HEK293: Human embryonic kidney 293
DPSCs: Dental pulp stem cells
qRT-PCR: Quantitative real-time polymerase chain reaction
IF: Immunofluorescence
KEGG: Kyoto Encyclopedia of Genes and Genomes
