# Supplementary material for: FGFR1 variants contributed to families with tooth agenesis
Source: Hum Genomics. 2023 Oct 13;17:93. doi: 10.1186/s40246-023-00539-8 (PMC10576343; doi:10.1186/s40246-023-00539-8)
Supplement: Supplementary file 1 — Additional file 1. Table S1. The top five candidate variants identified in family 1. Figure S1. Gene expression studies of FGFR1. Figure S2. Flow cytometric identification of hDPSCs surface markers shows stem cell characteristics. [file 40246_2023_539_MOESM1_ESM.docx]

*FGFR1* variants contributed to families with tooth agenesis

Siyue Yao^1,2,3#^, Xi Zhou^1,2#^, Min Gu^4#^, Chengcheng Zhang^1,2^, Oliver Bartsch^5^, Barbara Vona^6,7^, Liwen Fan^1,2^, Lan Ma^2*^, Yongchu Pan^1,2,8*^

^1^Department of Orthodontics, The Affiliated Stomatology hospital of Nanjing Medical University, Nanjing, China

^2^Jiangsu Province Key Laboratory of Oral Diseases, Nanjing Medical University

^3^The Affiliated Stomatology Hospital of Suzhou Vocational Health College, Suzhou 215000, China

^4^Department of Stomatology, Affiliated Third Hospital of Soochow University, The First People’s Hospital of Changzhou City, Changzhou City, Jiangsu Province, China, 213003

^5^Institute of Human Genetics, University Medical Centre of the Johannes Gutenberg University Mainz, Mainz, Germany

^6^Institute of Human Genetics, University Medical Center Göttingen, Göttingen, Germany

^7^Institute for Auditory Neuroscience and InnerEarLab, University Medical Center Göttingen, Göttingen, Germany

^8^Jiangsu Province Engineering Research Center of Stomatological Translational Medicine, Nanjing Medical University

^#^These authors should be considered joint first author

^*^Corresponding authors:

Yongchu Pan: Jiangsu Province Key Laboratory of Oral Diseases, Nanjing Medical University, 136 Hanzhong Road, Nanjing, 210029, China

Emails: panyongchu@njmu.edu.cn

Tel: +86-25-86862025; Fax: +86-25-86862823

Lan Ma: Jiangsu Province Key Laboratory of Oral Diseases, Nanjing Medical University, 136 Hanzhong Road, Nanjing, 210029, China

Emails: malan@njmu.edu.cn

authors’ Emails:

Siyue Yao: yaosiyue@njmu.edu.cn

Xi Zhou: zhouxi@stu.njmu.edu.cn

Min Gu: gumin106@163.com

Chengcheng Zhang: zhangchengcheng@stu.njmu.edu.cn

Oliver Bartsch: oliver.bartsch@mvz.unimedizin-mainz.de

Barbara Vona: barbara.vona@med.uni-goettingen.de

Liwen Fan: liwenfan@njmu.edu.cn

| Table S1. The top five candidate variants identified in family 1 | | | | | | | | | |  |  |  |  |  |
| --- | --- | --- | --- | --- | --- | --- | --- | --- | --- | --- | --- | --- | --- | --- |
| Chr | position | dbSNP | Gene | Variant | Type | Exon | gnomAD | Genotype reported in HGMD | Mutation Taster^1^ | | CADD^2^ | SIFT^­­­3^ | PolyPhen-2^4^ | Phenolyzer Score^5^ |
| 8 | 38287455 | rs773442656 | *FGFR1* | c.103G>A | Missense | Exon3 | 0.00002942 | heterozygous | 1.00 | | 21.30 | 0.14 | 0.02 | 0.41 |
| 11 | 75907604 | rs781536011 | *WNT11* | c.242G>A | Missense | Exon2 | 0.00003192 | heterozygous | 1.00 | | 31.00 | 0.01 | 0.51 | 0.09 |
| 12 | 26493155 | rs200691111 | *NFATC1* | c.415G>A | Missense | Exon5 | 0.0002921 | heterozygous | 1.00 | | 27.80 | 0.01 | 1.00 | 0.09 |
| 18 | 77211744 | — | *ITPR2* | c.7964C>T | Missense | Exon56 | — | heterozygous | 1.00 | | 24.10 | 0.01 | 0.08 | 0.06 |
| 20 | 57428331 | rs545306394 | *GNAS* | c.11G>A | Missense | Exon1 | 0.0001234 | heterozygous | 1.00 | | 23.50 | 0.01 | 0.63 | 0.05 |

^1^ The value indicates the effect of the variant on the protein sequence, the higher the value the more " deleterious ", indicating that the SNP has a high probability of causing structural or functional changes to the protein.

^2^ Higher values are more deleterious.

^3^ Deleterious (SIFT ≤ 0.05); Tolerated (SIFT > 0.05).

^4^ Probably damaging (≧0.909), P: possibly damaging (0.447≤pp2_hvar≤0.909); B: benign (pp2_hvar≤0.446).

^5^ The higher the score, the higher the correlation with the disease.


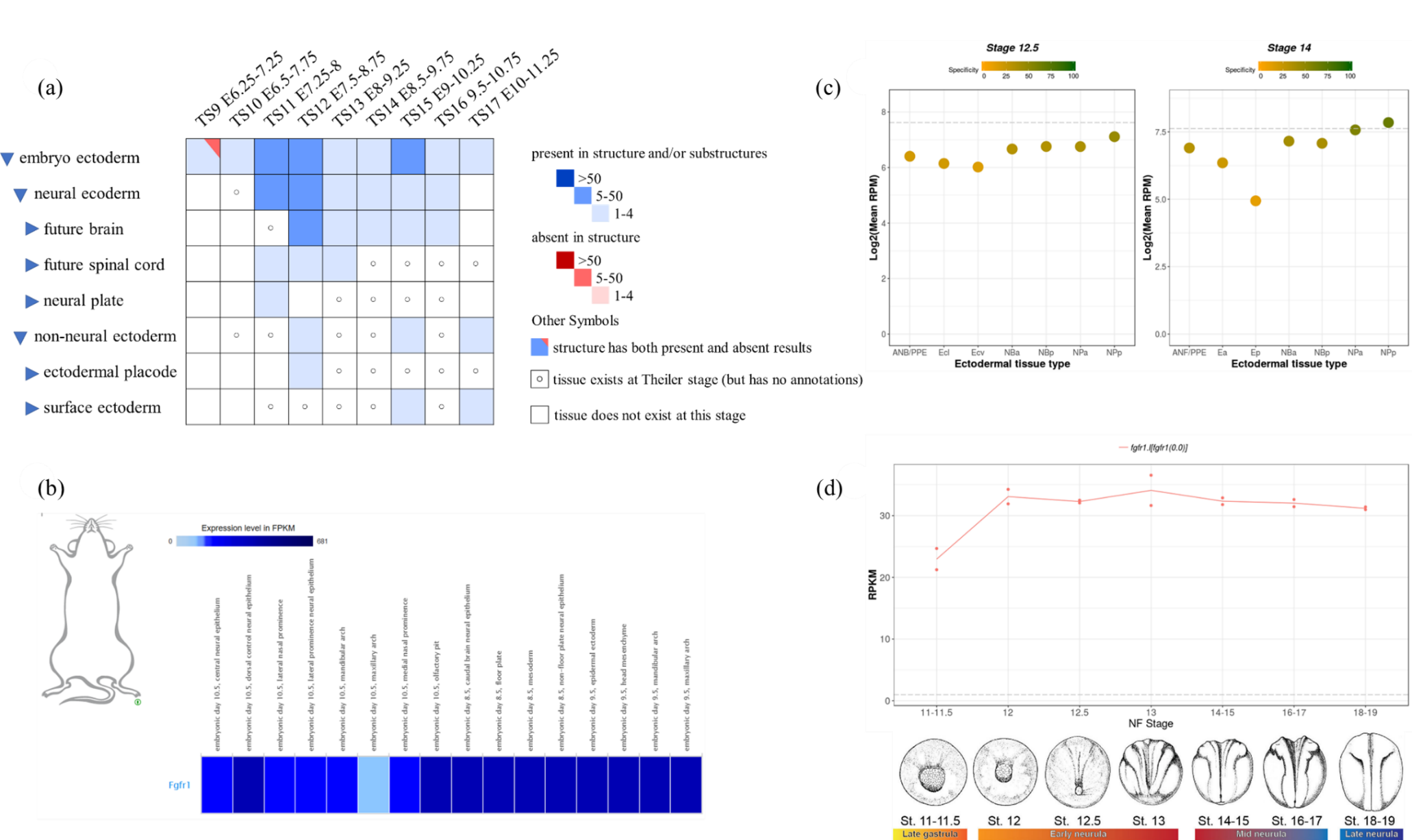


**Figure S1. Gene expression studies of *FGFR1*.** *FGFR1* expression during early embryonic development of mice (a, b) and *Xenopus laevis* frogs (c, d).


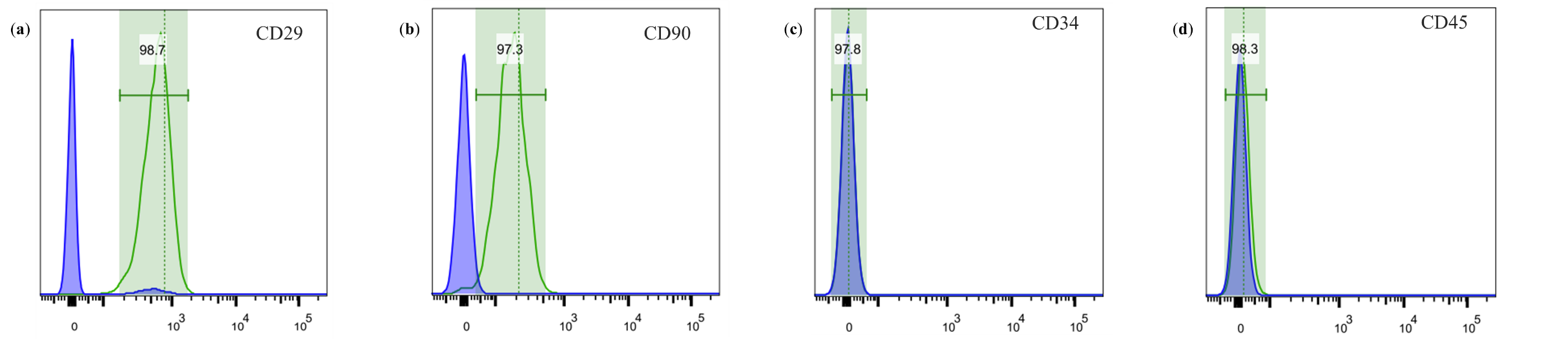


**Figure S2.** Flow cytometric identification of hDPSCs surface markers shows stem cell characteristics. (a-b) Flow cytometry demonstrated that DPSCs presented positive for CD29 and CD90. (c-d) Flow cytometry showed that DPSCs were negative for hematopoietic markers of CD34 and CD45.
